# Organ Abnormalities Caused by Turner Syndrome

**DOI:** 10.3390/cells12101365

**Published:** 2023-05-11

**Authors:** Sang Hoon Yoon, Ga Yeon Kim, Gyu Tae Choi, Jeong Tae Do

**Affiliations:** Department of Stem Cell and Regenerative Biotechnology, KU Institute of Technology, Konkuk University, Seoul 05029, Republic of Korea

**Keywords:** Turner syndrome, X monosomy, X chromosome inactivation, organ abnormalities

## Abstract

Turner syndrome (TS), a genetic disorder due to incomplete dosage compensation of X-linked genes, affects multiple organ systems, leading to hypogonadotropic hypogonadism, short stature, cardiovascular and vascular abnormalities, liver disease, renal abnormalities, brain abnormalities, and skeletal problems. Patients with TS experience premature ovarian failure with a rapid decline in ovarian function caused by germ cell depletion, and pregnancies carry a high risk of adverse maternal and fetal outcomes. Aortic abnormalities, heart defects, obesity, hypertension, and liver abnormalities, such as steatosis, steatohepatitis, biliary involvement, liver cirrhosis, and nodular regenerative hyperplasia, are commonly observed in patients with TS. The *SHOX* gene plays a crucial role in short stature and abnormal skeletal phenotype in patients with TS. Abnormal structure formation of the ureter and kidney is also common in patients with TS, and a non-mosaic 45,X karyotype is significantly associated with horseshoe kidneys. TS also affects brain structure and function. In this review, we explore various phenotypic and disease manifestations of TS in different organs, including the reproductive system, cardiovascular system, liver, kidneys, brain, and skeletal system.

## 1. Introduction

**Figure 1:**
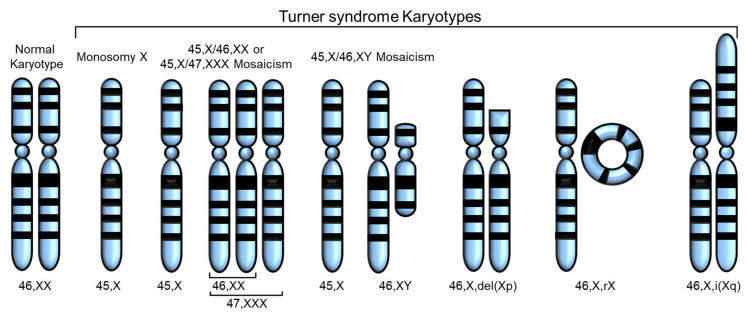
Various karyotypes of Turner syndrome (TS) modified from Huang et al. [8]. Partial or complete loss of the second sex chromosome results in TS. The most common karyotype in TS is monosomy X (45,X), and the others are 45,X/46,XX or 45,X/47,XXX mosaicism, 45,X/45,XY mosaicism, deletion of Xp or Xq, ring X (46,X,rX), and isochromosome Xq.

Turner syndrome (TS) is one of the most common disorders caused by chromosomal abnormalities, affecting approximately 1 in 2500 live female births. It is the only viable monosomy syndrome caused by partial or complete loss of one of the two sex chromosomes [1]. TS was first reported in 1938 by Henry H. Turner as a syndrome of infantilism, congenital webbed neck, and cubitus valgus, and Ford et al. found that the disease was caused by sex chromosomal abnormality in 1959 [2,3]. The most common karyotype in TS is 45,X, accounting for 40–50% of all cases of TS, whereas 45,X/46,XX or 45,X/47,XXX mosaicism account for 20–30%. The remaining cases include Y chromosome variants and X chromosome structural abnormalities, such as isochromosome Xq, deletion of Xp or Xq (which can occur as mosaicism), and ring X (which is always mosaic) [4] (Figure 1). Thus, in TS, only one X chromosome is normal and the others are absent or abnormal. The diagnosis of TS has traditionally relied on the clinical phenotype in addition to standard chromosomal analysis [5]. Total or partial loss of one of the two sex chromosomes affects biological pathways and networks [5], and, in some cases, *SHOX* gene defects have been linked to certain phenotypes of TS [6,7] (Table 1).

Individuals with TS are at an increased risk of endocrine diagnoses, including diabetes, thyroid and parathyroid disorders, celiac disease, and osteoporosis [23,24], as well as cardiovascular diseases, including arrhythmia, ischemic heart disease, hypertension, hyperlipidemia, and stroke. This is supported by the increased use of prescription drugs by patients with TS [25]. The 45,X karyotype is associated with the highest rates of morbidity and mortality, whereas the mosaic karyotype is associated with a low prevalence for cardiovascular, metabolic, renal, and reproductive phenotypes [26,27,28,29,30,31,32,33]. Despite ongoing research, no feasible treatment has been proposed owing to the severe effects of losing an entire chromosome and the numerous genes that are simultaneously affected [34]. In this review, we aim to summarize the symptoms and organ abnormalities observed in patients with TS and the consequences of X chromosome inactivation.

## 2. Fertility Problems

Infertility is one of the most common symptoms of TS, despite low rates of spontaneous pregnancies [31,35,36,37]. TS is accompanied by hypogonadotropic hypogonadism in almost all patients, leading to primary or secondary amenorrhea and infertility owing to premature ovarian failure (POF) (affecting approximately 95% of women with TS) and premature ovarian insufficiency [28,38,39]. Therefore, women with TS do not produce enough eggs or the necessary hormones to support pregnancy. The ovaries in a 45,X fetus appear to develop normally until birth; however, follicular atresia is induced by birth or early childhood [40]. Moreover, 5–20% of girls with TS retain enough follicles to permit spontaneous menarche, even if early menopause typically follows. Women with TS who have a mosaic karyotype, or experience spontaneous puberty, have follicles in one or both ovaries [39]. Furthermore, those with low levels of 45,X/46,XX mosaicism are less severely affected and have a high likelihood of experiencing spontaneous menstruation and pregnancy, although karyotype does not always predict phenotype [41,42,43]. Accelerated germ cell death is presumed to be the major mechanism causing germ cell depletion in patients with TS.

Reynaud et al. analyzed 10 aborted fetuses with TS and found that the number of germ cells in the genital ridge was similar to that in the control group up to 12 weeks of gestation, indicating normal migration of primordial germ cells in fetuses with TS [44]. However, differences were observed from 18 weeks of gestation, where germ cells were rarely detected, and completely absent at 25 weeks of gestation in fetuses with 45,X TS. Moreover, primordial and antral follicles were absent in fetuses with 45,X TS, although they were present in fetuses with TS with mosaicism. These studies suggest that folliculogenesis is severely impaired in ovaries of patients with TS, possibly owing to the loss of germ cells [44]. Additionally, the eggs from women with TS might be of poor quality, decreasing the chances of successful fertilization and pregnancy [45].

TS can also cause abnormalities in the structure and function of the uterus, affecting the implantation and growth of fertilized eggs [46]. Only about a quarter of people with TS have a fully developed uterus in size and shape, while most others have a slightly smaller uterus; about one-third have an immature form of the uterus. Notably, the difference in the size of the uterus between women with TS and those with a normal karyotype is not significant; however, on average, women with TS have a smaller uterine volume than those with a normal karyotype. The size of the uterus in individuals with TS can be influenced by various factors, including the age of the patient, duration of estrogen use, use of hormone replacement therapy (HRT), and type of estrogen medication administered. However, with appropriate and timely treatment, women with TS can achieve normal uterine development [46].

In addition, an imbalance in sex hormone levels affects the fertility of patients with TS. Women with TS showed 30–50% lower levels of androgens, including testosterone, free androgen index, androstenedione, and dehydroepiandrosterone sulfate, than those with a normal karyotype, but an increase in Follicle stimulating hormone (FSH), Luteinizing hormone (LH), and estrone sulfate levels up to twice the normal range [47]. High levels of FSH and LH during adolescence are linked to reduced ovarian function [48]. However, patients with TS showed a normal biphasic age pattern of reproductive hormones, with peak FSH and LH levels occurring at three months of age, followed by a subsequent decrease to minimal levels during mid-childhood and reactivation at puberty [48,49].

Pregnancy is rare among patients with TS and shows a high risk of miscarriage, stillbirth, and birth defects [50]. Only 2–5% of patients with TS become pregnant spontaneously, and approximately 3.8% of patients with TS have one or more live-born children [28,35]. Both natural and medically assisted pregnancies in patients with TS have a higher risk of adverse maternal and fetal outcomes than those in healthy women. For instance, 23–50% of women with TS have congenital heart disease, and pregnancy causes a 50% increase in cardiac output, making patients with TS susceptible to aortic dissection or rupture. As a result, the risk of death during pregnancy for patients with TS can reach up to 2% [51,52].

The *CSF2RA* (colony-stimulating factor 2 receptor alpha) gene located in the pseudoautosomal region 1 (PAR1) of the X chromosome is possibly involved in intrauterine lethality in fetuses with 45,X TS [21]. *CSF2RA* expression is downregulated in placental cells of females with 45,X TS compared with that in controls [13,22]. *USP9X* (located in Xp11.4) is a candidate for the failure of gonadal and oocyte development in TS [53]. *ZFX* (located on Xp21.3) and *USP9X* (located on Xp11.4) are possibly involved in ovarian failure in TS. In addition, patients with a loss of the *USP9X* region experience primary amenorrhea [54]. However, further studies are needed to investigate its role in TS.

Accordingly, women with TS who want to have children may opt for fertility care, such as in vitro fertilization (IVF) or egg donation. However, these treatments may be less effective in patients with TS owing to the various underlying fertility problems.

## 3. Heart and Cardiovascular Disease

Congenital and acquired heart defects and cardiovascular conditions are the leading cause of death in patients with TS, affecting about 25–50% of cases, with a higher incidence in those with 45,X karyotypes than in those with other TS variants [55]. Miyabara et al. conducted an autopsy of a 20-week-old fetus with 45,X karyotype and found that the wall of the aortic arch was much thinner than normal and that the number of smooth muscle cells and elastic fibers in the aorta was significantly reduced [56]. Anomalies of the coronary arteries are diverse and include many variants other than two arteries originating from aortic sinuses [57]. Many types of coronary artery anomalies have been reported in TS, especially in patients with bicuspid aortic valve (BAV) [58,59,60]. Although not all patients with TS have arch anomalies, aortic arch anomalies are common in TS owing to the complex embryological development of this vessel [61,62,63]. The most common anomalies include elongation of the arch and aberrant right subclavian artery [64,65]. Patients with TS and aortic arch anomalies are also at risk of developing aortic dilation, which could increase the risk of aortic dissection, occurring in 1–2% of patients with TS [66,67].

Aortic arch hypoplasia is another congenital aortic anomaly associated with TS and may vary in severity from mild aortic stenosis to severe transverse arch hypoplasia, interrupted aortic arch, or hypoplastic left heart syndrome [68]. Patients with TS are also prone to increased carotid artery thickness and arterial diameter, possibly owing to estrogen deficiency, which can be attenuated by estrogen hormone therapy [69,70,71]. Abnormalities of the venous system, such as hypoplasia of the portal vein system, are also observed in patients with TS, and vascular atrophy is involved in liver dysfunction [72].

Cardiovascular disorders caused by TS include early-onset hypertension, ischemia, and stroke. Hypertension is common in patients with TS, especially in children and adolescents, with an occurrence of approximately 21–40%. [73]. One possible cause of hypertension in TS is the coarctation of the aorta, and another possible cause is kidney dysfunction, which can lead to excessive retention of salt and water in the body and an increase blood pressure by increasing blood volume [74]. Furthermore, obesity and metabolic syndromes can also contribute to hypertension in patients with TS. Hypertension can be a risk factor associated with myocardial infarction, aortic dissection, ischemia, and stroke [75]. Therefore, ischemic heart disease and stroke are common symptoms in patients with TS [76]. As stroke is caused either by a blockage in the blood vessel supplying blood to the brain or by bleeding in the brain, patients with TS have an increased risk of stroke owing to the high incidence of vascular defects, hypertension, and heart disease [77].

Commonly observed congenital heart defects (CHD) in patients with TS are left-sided lesions, such as BAV, coronary artery anomalies, and congenital aortic arch anomalies. BAV, which occurs in up to 30% of TS cases, is the most common congenital malformation (fusion of right/left coronary cusps), compared with a 1–2% incidence in the general population [58,78,79]. BAV results from the failure of the two leaflets of the aortic valve to separate during embryogenesis and increases the risk of valvular and aortic pathologies, such as aortic insufficiency, aortic aneurysm, and aortic dissection [80]. Although the underlying link between TS and CHD is not fully understood, genes located on the X chromosome may play a role in the development of left-sided heart structures [81,82].

As males are affected three times more often than females, individuals with TS are approximately 60 times more likely than euploid females to have BAV [80]. The presence of BAV and the results of karyotyping in female infants serve as indicators for the earlier diagnosis of TS [55]. Aortic dissection is common in patients with TS and occurs even early in life. In a study, about half of 84 patients with TS were younger than 30 years of age [83,84]. Hypertension, BAV, and aortic stenosis, which are the common symptoms of TS, may accompany aortic dissection [84].

Heart arrhythmias, including tachycardia (fast heart rhythm) and bradycardia (slow heart rhythm), are also observed in patients with TS. Although tachycardia is a common phenomenon in patients with TS, bradycardia is rarely seen [85]. The most commonly seen arrhythmia in patients with TS is supraventricular tachycardia, and other types of arrhythmias in patients with TS include atrial fibrillation, atrial flutter, and sinus bradycardia [85,86]. Although the exact mechanisms underlying the increased risk of cardiac arrhythmias in patients with TS are not fully understood, structural abnormalities in patients with TS, such as aortic stenosis and BAV, can lead to changes in blood flow and increased stress on the heart muscle. Patients with TS also have longer QT intervals than normal women, which may be associated with tachycardia [87].

As abnormal extracellular matrix (ECM) composition induces aortic structural malformation, matrix metalloproteinases (MMPs, a degradation factor of ECM), and tissue inhibitors of matrix metalloproteinases (TIMPs, inhibitor of MMPs) are involved in aortic abnormalities [88]. Increased expression of MMPs and reduced expression of *TIMP1* and *TIMP3* can lead to the degradation of ECM components of the aortic wall, resulting in thinning of the aortic wall and enlargement of the diameter. These changes are implicated in the pathogenesis of various abnormal aortic morphogeneses, such as BAV and aortic aneurysms [88,89]. Therefore, hemizygous expression of *TIMP1* on the Xp locus in patients with TS may increase susceptibility to abnormal aortic morphogenesis. Decreased expression of *TIMP3*, a *TIMP1* paralogue on chromosome 22, can augment the risk for aortopathy and BAV [88]. In addition, *TIMP1* is hypermethylated, which suggests that this gene is epigenetically inactive in patients with TS [90]. Moreover, reduced expression of *TIMP1* and *TIMP3* was observed in the euploid population with BAV and aortopathy [89].

## 4. Liver Abnormalities

Although liver involvement is mostly asymptomatic in patients with TS, a wide range of abnormal phenotypes may be observed in the liver, including steatosis, steatohepatitis, liver cirrhosis, biliary involvement, and nodular regenerative hyperplasia (NRH) [91,92,93,94,95,96] (Table 2). Singh et al. reported that approximately twice the number of girls with TS showed liver enzyme elevation (alanine aminotransferase and aspartate aminotransferase) compared with normal controls [97]. These liver enzyme levels have clinical significance as girls with TS with elevated liver enzyme levels are more likely to be diagnosed with liver disease [97]. For example, hypertransaminasemia is common in patients with TS and is typically associated with hepatic steatosis, which can also be caused by other factors, such as diabetes mellitus and dyslipidemia [98]. In addition, women with TS with elevated liver enzymes are overweight and exhibit high levels of cholesterol, triglycerides, apolipoproteins A and B, and gamma-glutamyl transferase [99]. Excessive body weight is a common cause of liver disease in patients with TS [99,100]. Patients who are overweight (>25 kg/m^2^), as defined by body mass index (BMI) values, frequently experience insulin secretion disorders and diabetes mellitus. However, increased weight and BMI in patients with TS are not necessarily estrogen-related. Moreover, the lack of estrogen or GH (Growth Hormone) treatment is not the primary cause of the increase in liver enzymes. Blackett et al. observed that weight and BMI characteristics of normal girls did not correlate with those in patients with TS, as shown in statistical graphs of weight and BMI for patients according to their age [101]. Wójcik also suggested that 34% of adolescent patients with TS exhibit enzyme abnormalities, which do not always correspond to obesity [102].

Patients with TS have a relatively high incidence of non-alcoholic fatty liver disease (NAFLD), which can range from benign steatosis to steatohepatitis that may progress to fibrosis and cirrhosis. Accordingly, patients with TS have a five-fold increased risk of liver cirrhosis compared with normal controls [96]. In patients with TS without changes in liver architecture, the presence of mild-to-moderate portal fibrosis, as well as microvascular or macrovascular steatosis and inflammatory infiltration, was observed [72]. However, patients with TS with architectural changes in the liver mainly suffer from periductal fibrosis, along with fibrosis of the trachea invading the interlobular bile duct and some septal ducts. These patients also exhibit a high frequency of portal hypertension, aortic bicuspid, coarctation, and stenosis [72]. Severe structural changes in the liver, such as NRH, multiple focal nodular hyperplasia (FNH), and cirrhosis have also been observed in individuals with TS [72,104]. NRH and FNH are benign liver conditions characterized by the transformation of normal liver tissue into multiple small clusters (nodules) of regenerating liver cells. Although the cause of these hyperplasias is not well understood, it is possibly related to abnormal blood vessels in the liver. Thus, hyperplasia is a type of vascular disorder. Cirrhosis, a vascular abnormality commonly seen in patients with TS, is a leading cause of chronic liver disease [105]. Moreover, patients with TS are prone to developing various types of biliary involvement, including primary sclerosing cholangitis (PSC), which is associated with an increased prevalence of inflammatory bowel disease. Abnormal angiogenesis may be the underlying mechanism for the abnormal development of the bile duct in patients with TS [107]. In addition, primary biliary cirrhosis (PBC), characterized by the destruction of bile ducts, is more common in patients with TS than in normal women [109].

Functional abnormalities of the liver in patients with TS are more common in elderly women than in young girls with TS. In several case studies on adults, abnormal liver functional changes occurred in 36% of patients with abnormal levels of one or more liver enzymes and in 23% of the remaining patients during follow-up [103,108]. While patients with TS are more likely to be overweight or obese owing to abnormal body proportions, liver functional differences in overweight patients with TS are similar to those in obese patients without TS. Liver lesions in patients with TS are likely caused by fat accumulation, body weight, and BMI increase with age, rather than as a direct result of TS [102]. Functional changes in the liver in patients with TS may be caused by estrogen replacement therapy owing to the role of estrogen receptors in liver lipid homeostasis [102,105,136,137,138]. Although fatty liver disease is closely related to TS, it is a prevalent feature in the general population, and increased alcohol intake can promote its formation [93,99,139,140]. Therefore, the reason for the increase in liver enzymes or abnormal liver function in patients with TS is not specific to TS and requires critical examination of the cause.

## 5. Kidney Abnormalities

Kidney abnormalities are common in patients with TS, with a prevalence of 33–70%, and include kidney and urinary tract anomalies, such as abnormal ureter structure leading to urine regurgitation, horseshoe kidney (kidney fusion), renal aplasia, duplex collecting system, single unilateral kidney, and formation of cilia and cysts in the kidney [111,112,113,117] (Table 2). The most frequently reported renal anomaly is the horseshoe kidney, which occurs in 20–45% of patients with TS, whereas it is observed in less than 3% of the general population [141,142]. Horseshoe kidney is caused by the fusion of the two kidneys, forming a U-shaped structure. While patients with TS with horseshoe kidney may be asymptomatic during childhood, they may experience recurrent urinary tract infections and kidney stones in the later stages of the condition [112,115,116,119]. The incidence of renal malformations is significantly higher in patients with TS with a non-mosaic 45,X karyotype than in those with mosaicism, probably owing to lymphatic retention and organ system compression [116]. Hypertension can also be caused by renal malformations besides aortic stenosis and intrarenal vascular changes in patients with TS [116]. Other rare cases of malformations include rotation and postural abnormalities, severe and mild hydronephrosis, and unilateral/bilateral overlap collector type.

In TS, congenital anomalies of the kidney and renal-urinary tract (CAKUT) can manifest as hemiplegic, neoplastic, and polycystic kidneys. While most patients with horseshoe kidneys have normal kidney function, renal hypoplasia may lead to impaired renal function. X-structural abnormalities were observed in 68.7% of patients with a non-mosaic 45,X karyotype and in 9.0% of patients with a 45,X mosaic karyotype. In those with 45,X monosomy, 45,X with mosaicism, and X-structural abnormalities, the CAKUT incidence was 11.5%, 7.4%, and 25.0%, respectively, indicating a reduced ability to form kidneys with non-mosaic X chromosome abnormalities [112].

Collectrin, Amino Acid Transport Regulator (*CLTRN*) was found in patients with TS with renal phenotypic abnormalities. However, no specific nucleotide changes were identified that could lead to gene mutations. *CLTRN*, located in Xp22.2, is a dosage-sensitive X-linked gene related to TS and kidney function, such as amino acid transport in the kidney [143,144]. Recently, Pajenda et al. suggested that the excretion of *CLTRN* in urine could be a biomarker for acute kidney injury, as it decreases during kidney injury [144]. Therefore, evaluating *CLTRN* gene expression is worth considering in patients with TS with an abnormal renal phenotype. In other words, a direct link between the gene and renal malformation in patients with TS cannot be proven; however, TS is expected to have an indirect effect [114].

## 6. Skeletal Abnormalities and Short Stature

Girls with TS often suffer from reduced bone density and delayed bone formation owing to estrogen deficiency during adolescence [126,145] (Table 2). Osteopenia or osteoporosis are identified as common factors for problems in bone formation [126,127,128,129,130]. Growth retardation of the joints of the finger bones was also noticeable in patients with TS compared with normal controls. This difference in bone formation between patients with TS and normal controls is minor until the age of 10 years, but becomes more significant during puberty [125].

Bone density analysis showed decreased bone density in patients with TS in various areas [120,121]. For example, bone mineral apparent density (BMAD) in patients with TS was significantly lower in the femoral neck—an area of predominantly cortical bone—than in normal controls [120,122]. In addition, proximal radius and cortical volumetric bone mineral density (vBMD) exhibited a decreasing trend in cortical thickness [120,123]. BMD was maintained predominantly in trabecular bone, and BMD in the lumbar spine, an area rich in trabecular bone, was not significantly different between TS and control groups [120]. Women with TS also showed low BMAD in the cortical and trabecular bone of the forearm [124], and the width of the ultradistal radius (predominantly in the trabecular bone) was reduced [122].

Women with TS have a >60% chance of having facial skeletal malformations, which include micrognathia, outer corners of the eyes and epicanthic folds, high arched palate, and low-set ears [131,132]. Some researchers speculate that these malformations are associated with reduced expression of the *SHOX* gene or other genes located in the PAR1 of the X chromosome [21,146]. Hearing loss is common in patients with TS (63–70%) owing to chronic or recurrent otitis media, sensorineural hearing loss, underdeveloped semicircular canals, and deformity of the auricle [132,133,134,135]. These are caused by phenotypic disorders of cartilage and bone formation of the face and ears.

Since abnormal bone formation in patients with TS may be caused by estrogen deficiency, early Hormone replacement therapy (HRT) using estrogen (estrogen replacement therapy; ERT) is recommended. The ERT in the early-start group (starting at the age of 18 years) showed high bone density formation [147,148,149]. ERT started after adulthood to suppress the rapid decrease in bone density may show an insignificant difference compared with the early-treatment group [145,150]. ERT is also effective in increasing uterine length and BMD [151]. While late ERT is effective for uterine growth and bone mass acquisition, early ERT is much more effective in ensuring sufficient bone mass [150,151].

Short stature is one of the most common characteristics in women with TS, and, when untreated, they are about 20 cm shorter than normal women [152]. Their growth pattern is also very different from the general population; they are typically born smaller and grow slowly until puberty; however, the growth velocity increases following puberty. Girls with TS typically lack a pubertal spurt, resulting in a significant drop in growth rate during puberty. However, unlike the general population, girls with TS do not experience epiphyseal closure, which allows for continued growth after puberty. Moreover, growth hormone treatment for increasing height is not very effective in girls with TS [153].

The short-stature homeobox (*SHOX*) gene is the most well-defined gene to explain the genetic mechanism of TS. The *SHOX* gene in humans is located at Xp22.3 and Yp11.3 and is highly expressed in bone morphogenetic tissues [154]. Therefore, *SHOX* haploinsufficiency is suggested to be the main cause of skeletal anomalies and short stature in patients with TS [155].

More severe skeletal deformation can be caused by homozygous *SHOX* deficiency, such as Langer mesomelic dysplasia, showing extremely short limbs, limb deformity, and short stature [155]. Other phenotypes related to *SHOX* observed in patients with TS include Madelung’s wrist deformity, forearm valgus, increased sitting height-to-height ratio, short fourth metacarpal, high arched palate, and micrognathia [9,10,11]. *SHOX* is also associated with height gain in Klinefelter syndrome (47,XXY), Triple X (47,XXX), and Double Y (47,XYY) [11].

Girls affected by TS usually require GH replacement therapy to alleviate hindered growth and improve metabolic health. Adequate GH levels can improve glucose homeostasis and metabolic syndrome, and aid in repartitioning fat mass to lean mass [156]. Although no clinically relevant abnormalities in the GH/insulin-like growth factor (IGF) axis have been reported in patients with TS [5], early recognition and timely investigation of TS can lead to better outcomes. GH treatment can potentially increase adult height in those who respond, in addition to initiating sex hormone replacement therapy. Some studies have reported a 5–8 cm increase in average height after GH treatment, with some showing even greater increases [113,157,158,159]. This can significantly improve the quality of life of individuals with TS.

## 7. Brain Abnormalities

While no visible brain abnormalities are apparent, structural, electrophysiological, cognitive, and psychosocial studies have reported differences between patients with TS and normal control. Reiss et al. suggested that the brain structure of women with TS could be distinguishable from that of age-matched controls [160] (Table 3). Several other studies also suggested that patients with TS had a small volume of cerebral hemispheres and an increased volume of cerebrospinal fluid and the fourth ventricle [161,162]. The size of gray and white matter mainly determines brain volume and size. When comparing patients with TS against controls, although not statistically significant, there was an increase in gray matter in the right superior temporal gyrus and left amygdala and an increase in white matter in the left superior temporal gyrus [163]. However, many reports also suggested that the brain structure was smaller than that of the control group. Compared with that of controls, in individuals with TS, there is a reduction in gray matter in various regions, including the right calcarine cortex, precentral region, supramarginal gyri, cuneus, lingual cortex, superior parietal, rostral anterior portion, pericalcarine, and postcentral and precuneus of the right hemisphere’s cingulate cortex, as well as a reduction in white matter in the entorhinal cortex, pars opercularis, frontal pole, and occipital lobe [164,165,166]. Most gray matter reductions are related to surface area reduction [164].

Patients with TS with maternally derived X chromosomes had less white matter in the occipital lobe and more gray matter in the cerebellum than normal controls. However, there was no significant difference between patients with TS with paternally derived X chromosomes and the control group [167]. Cutter et al. reported a patient with TS with a maternal X chromosome showing a significant decrease in gray matter volume in the caudate nuclei, extending to the posterior thalamus, and bilaterally expanding white matter volume in the temporal lobes [169]. Decreased gray matter in the caudate nuclei and white matter in the occipital lobe was found to be associated with patients with TS with a maternal X chromosome [167,169]. These findings suggest that patients with TS with a maternal X chromosome have more severe abnormal brain structures than those with a paternal X chromosome.

The structural differences in the brain of patients with TS may be related to the cognitive behavioral domain. Although TS is not usually associated with mental retardation, deficits in visual–spatial perception were shown in a relatively large number of patients with TS, whereas verbal and conception problems were sparsely observed [173,174]. Functional neuroimaging studies using positron emission tomography (PET) suggested a decrease in glucose metabolism in the parietal and occipital regions of the brain of patients with TS, indicating possible parietal hypometabolism [175,176]. Kuntsi et al. found a distinctive characteristic of patients with TS with a ring X who displayed a high risk of mental retardation and abnormality of brain structure [177]. In addition, activation of the right intraparietal sulcus decreased when patients with TS performed activities such as counting. Several cases have been reported where patients with TS suffer from a loss of mathematical ability and visuospatial sense [168,172,178,179]. These results suggest that the reduced gray matter in the right intraparietal sulcus may be related to the spatiotemporal loss in patients with TS. As a result, this reduction in the gray matter can cause a loss of sensory and computational abilities [171,180].

Electrophysiological studies found that elderly patients with TS exhibited different event-related potential compared with age-matched controls [181]. Tsuboi et al. suggested that some patients with TS had decreased alpha waves and increased beta waves, especially in women over the age of 35 years [182]. Girls and women with TS are less likely to engage in social activities and have an approximately 500-fold increased risk of autism [183,184]. However, this could be owing to environmental influences, such as low self-esteem caused by external differences and health problems [173].

## 8. Relevance to X Chromosome Inactivation and Escape Genes

During the early development of mammals, one of the two X chromosomes in females (XX) is randomly inactivated by X chromosome inactivation (XCI), by which the total amount of X-linked genes expressed in females becomes equivalent to that in males (XY) [185,186]. Normal female somatic cells contain one active X chromosome (Xa) and one inactive X chromosome (Xi), resulting in a XaXi state. If X-linked genes in the Xi are completely silenced, the removal of Xi from XaXi may not have a harmful effect on cells. However, in TS the complete or partial loss of the Xi leads to a myriad of abnormalities. This is because some genes located outside the condensed heterochromatin of the Xi can escape from inactivation and be expressed, leading to differences in the number of expressed X-linked genes between 46,XaXi and 45,Xa states. These genes that are expressed from the Xi are called escape genes [187,188]. Approximately 15% and 3% of X-linked genes in humans and mice, respectively, are escape genes [188]. Human X chromosomes have pseudoautosomal regions (PARs) that behave like autosomes where crossing over strictly occurs (Table 1). Genes within PARs on the X chromosome usually escape from XCI [155]. The PAR1 genes, including *SHOX*, play essential roles in the phenotypic traits associated with TS, including short stature, Madelung’s wrist deformity, and intellectual disabilities [9,10]. Variations in the expression of these genes may contribute to growth deficits or increased height in affected individuals. Decreased expression of *SHOX* contributes to growth deficits observed in patients with TS, whereas increased expression in Klinefelter syndrome (47,XXY), Triple X (47,XXX), and Double Y (47,XYY) is associated with increased height [11].

In addition, 12 genes (AKAP17A, ASMT, ASMTL, CD99, CD99P1, CRLF2, CSF2RA, DHRSX, FABP5P13, GTPBP6, IL3RA, PLCXD1, PPP2R3B, P2RY8, SHOX, SLC25A6, XG, and ZBED1) located outside the PAR1 region have a single functionally Y homolog and are broadly expressed in human tissues [12]. USP9X genes on the X chromosome could evade Xi and be expressed in both human adult and embryonic tissues [53]. Quilter et al. found that the expression of escape genes, USP9X and ZFX, was associated with immune cell development, oocyte growth, and ovarian development [54].

Variants of the *KDM6A* gene, known to escape XCI, are also associated with Kabuki syndrome, a multisystem syndrome with TS-like phenotypic traits, such as growth delay, short stature, varying degrees of intellectual disabilities, skeletal and renal abnormalities, and congenital heart defects [189,190]. *RPS4X* and *RSPS4Y* are also considered dosage-sensitive genes, and several studies reported *RPS4X* downregulation in TS [17,18,19]. Wang et al. identified 25 upregulated and 60 downregulated genes in patients with TS compared with those in normal women and found five genes, including *CD99, CSF2RA, MYL9, MYLPF*, and *IGFBP2,* possibly involved in the pathogenesis of TS [20]. In addition, epigenetic mechanisms, such as DNA methylation, are also involved in the etiology of TS [191]. However, further studies are required to understand the correlation between escape genes and TS.

## 9. Conclusions

The primary cause of TS is the haploinsufficiency of genes located on the X chromosome. Complete or partial loss of one of the two X chromosomes contributes to the insufficiency of X-linked genes. Notably, female mice with only one X chromosome exhibit minor external symptoms and do not exhibit severe TS-like symptoms [192]. This observation could be attributed to the fact that only 3% of genes escape XCI in mice, compared with 15% in humans. Deletions on the X chromosome that cause TS usually occur at the termini of the qX and pX regions, known as the PARs, which typically contain genes that escape XCI [155]. The PAR1 genes, including *SHOX*, play essential roles in the phenotypic traits associated with TS, including short stature, wrist deformity, and intellectual disabilities [9,10]. Other X-linked genes, such as *CSF2RA, ZFX*, and *USP9X*, play a role in immune cell development, oocyte growth, and ovarian development. These genes are also associated with abnormal phenotypes in various tissues in individuals with TS [21,54].

TS can lead to abnormalities in many tissues and organs, including the ovaries, uterus, heart, cardiovascular system, liver, kidneys, skeletal system, and brain. Additionally, individuals with TS may exhibit various phenotypes, such as short stature, diabetes, thyroid and parathyroid disorders, celiac disease, hypertension, arrhythmia, ischemia, hyperlipidemia, stroke, and osteoporosis [23,24]. The symptoms of TS are very complex and do not manifest as a single symptom. For example, vascular anomalies are associated not only with the heart but also with the kidney, liver, and other tissues. Problems with bone formation can lead not only to short stature and osteoporosis, but also to facial abnormalities and hearing impairment. While HRT may alleviate some symptoms of TS, there is currently no fundamental cure available. Therefore, further research is needed to explore methods for substituting the function of deleted genes and compensating for haploinsufficiency to achieve a fundamental treatment for TS.

## Figures and Tables

**Table 1:** Genes associated with Turner Syndrome (TS).

Gene	Location	Expression in TS	Associated Phenotype	Reference
*SHOX*	Xp22.33 and Y chromosome (PAR1)	Decreased expression	Short stature, Madelung wrist deformity, Intellectual disabilities	[9,10,11,12,13]
*ARSD, ARSE, ARSF*	Xp22.3	Loss owing to contiguous gene deletion syndrome	Chondrodysplasia punctata	[14,15,16]
*STS*	Xp22.31	Loss owing to contiguous gene deletion syndrome	X-linked ichthyosis	[13,14,15,16]
*GPR143*	Xp22.2	Loss owing to contiguous gene deletion syndrome	Ocular albinism type I	[14,15,16]
*ANOS1*	Xp23.3	Loss owing to contiguous gene deletion syndrome	Kallmann syndrome	[14,15,16]
*RPS4X*	Xq13.1	Downregulation	N/A	[13,17,18,19]
*CD99*	X and Y chromosomes (PAR1)	Downregulation	N/A	[13,20]
*CSF2RA*	X and Y chromosomes (PAR1)	Downregulation	N/A	[13,20,21,22]
*MYL9*	20q11.23	Downregulated	N/A	[20]
*MYLPF*	16p11.2	Downregulated	N/A	[20]
*IGFBP2*	2q35	Downregulated	N/A	[20]

**Table 2:** Liver, kidney, and skeletal symptoms and prevalence in patients with TS.

Organ	Symptom	Prevalence Rate	Reference
Liver	Steatosis	33.3% of all patients with TS (no changes in liver structure)	[72,96,98]
	Steatohepatitis	4.8–12% of all patients with TS	[103]
	Liver cirrhosis	6.7–16.5% of all patients with TS (20% of patients with liver structural changes)	[72,95,98,104,105,106]
	Bile stasis, biliary involvement	43.7% of all patients with TS	[91,92,94,107]
	Nodular regenerative hyperplasia (NRH)	33% of all patients with TS (60% of liver structure change group)	[72,98,104]
	Liver enzyme elevation (alanine aminotransferase and aspartate aminotransferase)	20% of all patients with TS	[93,97,100,103,108]
	Higher total cholesterol, triglycerides, and apolipoproteins a and b	59% of all patients with TS (36% of patients were initially high, 23% of patients were added as a result of follow-up for 5 years)	[99]
	Non-alcoholic fatty liver disease (NAFLD)	36.7% of all patients with TS (64.7% of group patients without changes in liver structure)	[72,96]
	Moderate portal fibrosis	50% of all patients with TS (88.2% of group patients without changes in liver structure)	[72]
	Periductal fibrosis	70% of all patients with TS (80% of patients in the liver structure change group, 76.4% of the group without liver structure change)	[72]
	Bile duct abnormalities	66.6% of all patients with TS (95.2% of patients with periductal fibrosis)	[72]
	Portal hypertension	13% of all patients with TS (40% of liver structure change group patients)	[72,96]
	Aortic bicuspid, coarctation, stenosis	20% of all patients with TS (50% of liver structure change group patients)	[72]
	Multiple focal nodular hyperplasia (FNH)	6.7% of all patients with TS (20% of patients with liver structural changes)	[72,104]
	Primary sclerosing cholangitis (PSC)	N/A	[107,109]
	Primary biliary cirrhosis (PBC)	78% of all patients with TS	[109,110]
Kidney	Horseshoe kidneys of different sizes	7–29% (13.5% of patients with TS had renal abnormalities)	[111,112,113,114,115,116,117]
	Renal aplasia	3% of all patients with TS	[113]
	Simple cilia and cysts	16% of all patients with TS	[118]
	Hydronephrosis	17.5% of all patients with TS	[117]
	Urinary tract infections and kidney stones	N/A	[116,119]
Skeletal	Reduced bone density	N/A	[120,121,122,123,124]
	Delayed bone formation	N/A	[122,125]
	Osteopenia or osteoporosis	N/A	[126,127,128,129,130]
	Face skeletal malformations (including micrognathia, outer corners of the eyes and epicanthic folds, high-arched palate, and low-set ears)	More than 60% of all patients with TS	[131,132]
	Hearing loss	63–70% of all patients with TS	[132,133,134,135]
	Sensorineural hearing loss (SNHL)	9–63% of all patients with TS	[132,134]
	Middle ear disease	91% of all patients with TS	[134]

**Table 3:** Abnormal brain structures in patients with TS.

Abnormal Structure		Site of Occurrence	Reference
More than normal controls	Gray matter	Right superior temporal gyrus	[163]
		Between the cerebellum	[167]
		Inferior temporal	[164]
		Superior temporal	[164]
		Subcortical	[164]
		Left amygdala	[168]
	White matter	Left superior temporal gyrus	[163]
		Temporal lobes	[169]
		Superior temporal	[164]
		Superior frontal	[164]
		Precentral	[164]
		Right parahippocampal cortex	[170]
		Right superior temporal gyrus	[170]
		Left Heschl’s gyrus	[170]
		Left middle and superior temporal gyri	[165]
Lesser than normal control	Gray matter	Symmetrical location of the right intraparietal sulcus	[171]
		Precentral	[165,166]
		Caudate nuclei	[169]
		Postcentral	[164,165]
		Supramarginal gyri	[165]
		Cuneus	[164]
		Lingual gyrus	[164]
		Pericalcarine	[164]
		Superior parietal	[164]
		Rostral anterior portion of the cingulate cortex	[164]
	White matter	Occipital lobe	[167]
		Pericalcarine	[164]
		Postcentral	[164]
		Precuneus	[164]
		Entorhinal cortex	[164]
		Pars opercularis	[164]
		Frontal pole	[164]
		Rostral anterior portion of the cingulate cortex	[164]
		Surface area	[164]
		Average cortical thickness	[164]
		Parietal lobe	[164,167,172]
		Hippocampus	[161,168]
		Caudate	[161]
		Lenticular	[161]
		Thalamic nuclei	[161]
		Parieto-occipital brain matter	[161]
		Superior parietal	[172]
		Postcentral gyri	[172]
		Calcarine cortex	[170]
		Lingual cortex	[170]
		Precentral gyrus	[170]
		Middle temporal gyrus	[170]
		Left frontal inferior operon	[170]
		Left frontal inferior trigonal	[170]

## Data Availability

Not applicable.
